# American Female Doctors

**Published:** 1861-07

**Authors:** 


AMERICAN FEMALE DOCTORS.—"Some time ago the visitors of M. Piorry's clinique, in La Charité, were amazed to see among the crowd of students who always throng to the wards of this most estimable and original professor, two American ladies, thickly veiled, who had crossed the Atlantic for the express purpose of seeing and admiring the inventor of the plessimeter, and the science of plessimetry, as exercised by his own hands. M. Piorry, who is, above all, Frenchman and *galant-homme*, paid the greatest attention to the ladies, and showed them a beautiful case of splenomacrosy, a remarkable instance of bronchosyphiosis, an interesting patient affected with enterocarciny, and an extraordinary case of angitromemphaxia. The ladies seemed to be quite familiar with the professor's wonderful nomenclature, which it is the great object of his life to introduce into all systems of pathology of the civilized and uncivilized world."—*Medical Times and Gazette.*

